# Microbial transformation of ginsenoside Rb1, Re and Rg1 and its contribution to the improved anti-inflammatory activity of ginseng

**DOI:** 10.1038/s41598-017-00262-0

**Published:** 2017-03-10

**Authors:** Shanshan Yu, Xiaoli Zhou, Fan Li, Chunchun Xu, Fei Zheng, Jing Li, Huanxi Zhao, Yulin Dai, Shuying Liu, Yan Feng

**Affiliations:** 1 State Key Laboratory of Microbial Metabolism, School of Life Sciences and Biotechnology, Shanghai Jiao Tong University, Shanghai, 200240 China; 2 Jilin Ginseng Academy, Changchun University of Chinese Medicine, Changchun, 130117 China; 3 College of Basic Medical Sciences, Jilin University, Changchun, 130021 Jilin China; 4 School of Life Sciences, Northeast Normal University, Changchun, 130024 China

## Abstract

Microbial transformation of ginsenosides to increase its pharmaceutical effect is gaining increasing attention in recent years. In this study, *Cellulosimicrobium* sp. TH-20, which was isolated from soil samples on which ginseng grown, exhibited effective ginsenoside-transforming activity. After protopanaxadiol (PPD)-type ginsenoside (Rb1) and protopanaxatriol (PPT)-type ginsenosides (Re and Rg1) were fed to *C*. *sp*. TH20, a total of 12 metabolites, including 6 new intermediate metabolites, were identified. Stepwise deglycosylation and dehydrogenation on the feeding precursors have been observed. The final products were confirmed to be rare ginsenosides Rd, GypXVII, Rg2 and PPT after 96 h transformation with 38–96% yields. The four products showed improved anti-inflammatory activities by using lipopolysaccharide (LPS)-induced murine RAW 264.7 macrophages and the xylene-induced acute inflammatory model of mouse ear edema. The results indicated that they could dramatically attenuate the production of TNF-α more effectively than the precursors. Our study would provide an example of a unique and powerful microbial cell factory for efficiently converting both PPD-type and PPT-type ginsenosides to rare natural products, which extends the drug candidates as novel anti-inflammatory remedies.

## Introduction

Ginseng (*Panax ginseng*), one of the most well-known herbal medicines in the world, has gained increasing attention in the treatment of cardiovascular disease, diabetes and central nervous diseases^1–3^. Ginseng use is popular given its prominent effects on inflammation, which is the body’s response to injury and infection^4^. Ginsenosides are regarded as the main active components of ginseng. To date, more than 180 ginsenosides have been identified from ginseng. Ginsenosides can be divided into two groups: PPD-type and PPT-type ginsenosides according to their structures. PPD-type and PPT-type ginsenosides share a common dammarane triterpenoid structure. Rb1, Rd, Rc and Rb2 are PPD-type ginsenosides with sugar moieties attached to the β-OH at C-3 and/or C-20 in the aglycon PPD. Ginsenosides Re and Rg1 are PPT-type ginsenosides with sugar moieties linked to the α-OH at C-6 and/or β-OH at C-20 in the aglycon PPT. These ginsenosides differ in sugar types, numbers and attachment positions. Moreover, ginsenoside Rb1, Rb2, Rc, Re and Rg1 are major ginsenosides that account for more than 80% of total ginsenosides. In addition to major ginsenosides, rare ginsenosides with minimal levels in ginseng continue to be identified^5^. Transformation of major ginsenosides *in vivo* or *in vitro* can generate the formation of rare ginsenosides and leads to structural changes in the type of dammarane, the number of sugar moieties, and the substituent groups^6–8^. Furthermore, emerging evidences have demonstrated that these structural changes affect their pharmaceutical activities^9^. Wang *et al*. observed that transformation of ginsenosides by artificial gastric juice increase cytotoxicity toward cancer cells^10^. Kim *et al*. reported that thermal deglycosylation of ginsenoside Rd enhances the anticancer activity of ginsenoside^11^. Hence, these findings imply that structural changes of certain ginsenosides by transformation are significantly related to their improved pharmaceutical activities.

Transformation of ginsenosides mainly occurs via deglycosylation at the sugar moieties. Therefore, previous studies have focused on converting major glycosylated ginsenosides to deglycosylated ginsenosides^12^. Various deglycosylation methods, including heat treatment^13^, mild acid hydrolysis^14^, enzymatic hydrolysis^15, 16^, and microbial transformation^17, 18^ were reported. Among the methods, microbial transformation and enzymatic deglycosylation were the most useful given high substrate specificity, lower byproducts and high production yields. Moreover, microbial transformation present unique advantages as its complex enzyme system can enrich reaction types and provide the possibility of obtaining novel ginsenosides, which may possess important pharmaceutical activities. Thus, research on the development of microbial transformation methods for increasing the pharmaceutical effect by structural modification of ginsenosides is gaining more attention.

As mentioned above, the anti-inflammatory effects of major ginsenosides including Rb1, Re and Rg1 have been reported^19^. Moreover, recent research indicates that structural modification of the major ginsenosides may contribute to improved anti-inflammatory activity. For example, Joh *et al*. reported that ginsenoside Rb1 was transformed to compound K by gut microbiota. Compound K was more effective and inhibited the production of pro-inflammatory cytokines more potently compared with Rb1^20^. Similarly, Lee *et al*. reported that 20(S)-protopanaxatriol, which is metabolized from ginsenoside Re, exhibited more potent anti-inflammatory effect compared with ginsenosides Re^21^. Kim *et al*. observed that 20(S)-protopanaxatriol is metabolized from ginsenoside Rg1 via ginsenosides Rh1 and F1 after oral administration, and these metabolites may ameliorate inflammatory disease, such as colitis, by inhibiting the binding of LPS to TLR4 on peritoneal macrophages and restoring Th17/Treg cell balances^22^. To date, only a few reports were available, and no ginsenosides have been developed as anti-inflammatory drugs. The research on ginsenosides with anti-inflammatory activity remains in the initial stage. Hence, transformation of major ginsenosides Rb1, Re and Rg1 may explore potential agents for anti-inflammatory therapies.

In this study, *Cellulosimicrobium* sp. TH-20 could utilize PPD-type (Rb1) and PPT-type (Re and Rg1) ginsenosides for rare ginsenosides production. The transformation process and the metabolites were qualitatively and quantitatively investigated by HPLC and LC-MS analysis. Moreover, the anti-inflammatory activities of the 4 final products and their precursors were assessed by using lipopolysaccharide (LPS)-induced murine RAW 264.7 macrophages and the xylene-induced acute inflammatory model of mouse ear edema. Our study would provide an example of a unique and powerful microbial cell factory for developing novel anti-inflammatory remedies.

## Results and Discussion

### Isolation and characterization of strain TH-20

In order to screen appropriate microbial strains for ginsenosides production, soil samples from ginseng field were collected. More than 20 microorganisms were isolated on ginseng agar medium plates. After major ginsenosides including Rb1, Rb2, Rc, and Rg1 were fed to these isolates, the transformation products were determined through thin layer chromatography (TLC) analysis. Ten isolates were confirmed to exhibit ginsenoside-transforming activities (Supplementary Fig. S1). Compared to other isolates, strain TH-20 exhibited unique and effective activities to transform both PPD-type and PPT-type ginsenosides.

The strain TH-20 was identified by morphological observation, biochemical characteristics and phylogenetic analysis. Cells were 1 μm wide and 1.8- to 4 μm long, Gram positive, rod-shaped, flagellated and non-sporulating short rods as assessed by scanning electron microscope (Supplementary Fig. S2). Colonies were yellow and irregular. Cells were positive for mannose, lactose, fructose, sucrose, arabinose, galactose, xylose, maltose, trehalose, sorbose, catalase, oxidase, nitrate reduction, gelatin liquefaction, hydrolysis of starch, production of H_2_S and indole but negative for rhamnose, raffinose, ribose, arginine dihydrolase and urease. Bergey’s Manual of Determinative Bacteriology was used to identify the strain according to the morphological characteristics and physiological and biochemical reactions, the results showed that strain TH-20 was gram positive and non spore bacterial and closed to *cellulomonas* sp. Phylogenetic analysis based upon the 16 S rDNA gene sequence of strain TH-20 indicated that the strain was actually grouped in the *Cellulosimicrobium* species (Supplementary Fig. S3). And the highest degree of 16 S rDNA gene identity was 99% with the sequence from *Cellulosimicrobium* sp. H2.

### Qualitative analysis of the biotransformation process by HPLC and LC-MS analysis

**Figure 1:**
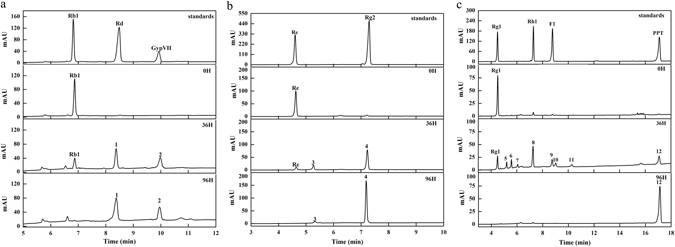
HPLC analysis of ginsenoside Rb1 (**a**), Re (**b**), and Re (**c**) during biotransformation process by using *Cellulosimicrobium* sp. TH-20.

The technique of HPLC was performed to estimate the qualitative analysis on different ginsenosides during biotransformation process by *Cellulosimicrobium* sp. TH-20. As shown in Fig. 1, transformation of ginsenoside Rb1, Re and Rg1 yielded twelve metabolites (metabolites 1–12) at 36 h. The six metabolites were identified as ginsenoside Rd (1), gypenoside XVII (2), ginsenoside Rg2 (4), ginsenoside Rh1 (8), ginsenoside F1 (9) and PPT (12) through comparison with ginsenoside standards by retention time. The other six ones (metabolites 3, 5, 6, 7, 10, and 11) were intermediates with new structures. Moreover, rare ginsenoside Rd (1), gypenoside XVII (2), ginsenoside Rg2 (4), and PPT (12) were the final products after 96-hour transformation. Structures of intermediates 3, 5, 6, 7, 10 and 11 were determined via rapid resolution liquid chromatography coupled with quadruple-time-of-flight mass spectrometry (RRLC-Q-TOF MS) and ultrahigh-performance liquid chromatography combined with Q-Exactive Orbitrap hybrid quadrupole-Orbitrap mass spectrometry (UHPLC-Q-Exactive Orbitrap HRMS) (Supplementary Figs S4–S9). They were identified to be 20-C-4-carbonyl-glycuronic Re (3), 20-C-4-carbonyl-glycuronic Rg1 (5), 6-C-4-carbonyl-glycuronic Rg1 (6), 6, 20-C-4-carbonyl-glycuronic Rg1 (7), 6-C-4-carbonyl-glycuronic Rh1 (10), and 20-C-4-carbonyl-glycuronic F1 (11). From the structural analysis and the current state of knowledge regarding the biotransformation mechanism, it is concluded that deglycosylation and dehydrogenation occurred in the biotransformation process of ginsenosides Rb1, Re, and Rg1 with *Cellulosimicrobium* sp. TH-20.

### Biotransformation pathways of Rb1, Rb2 and Rc using *Cellulosimicrobium* sp. TH-20

**Figure 2:**
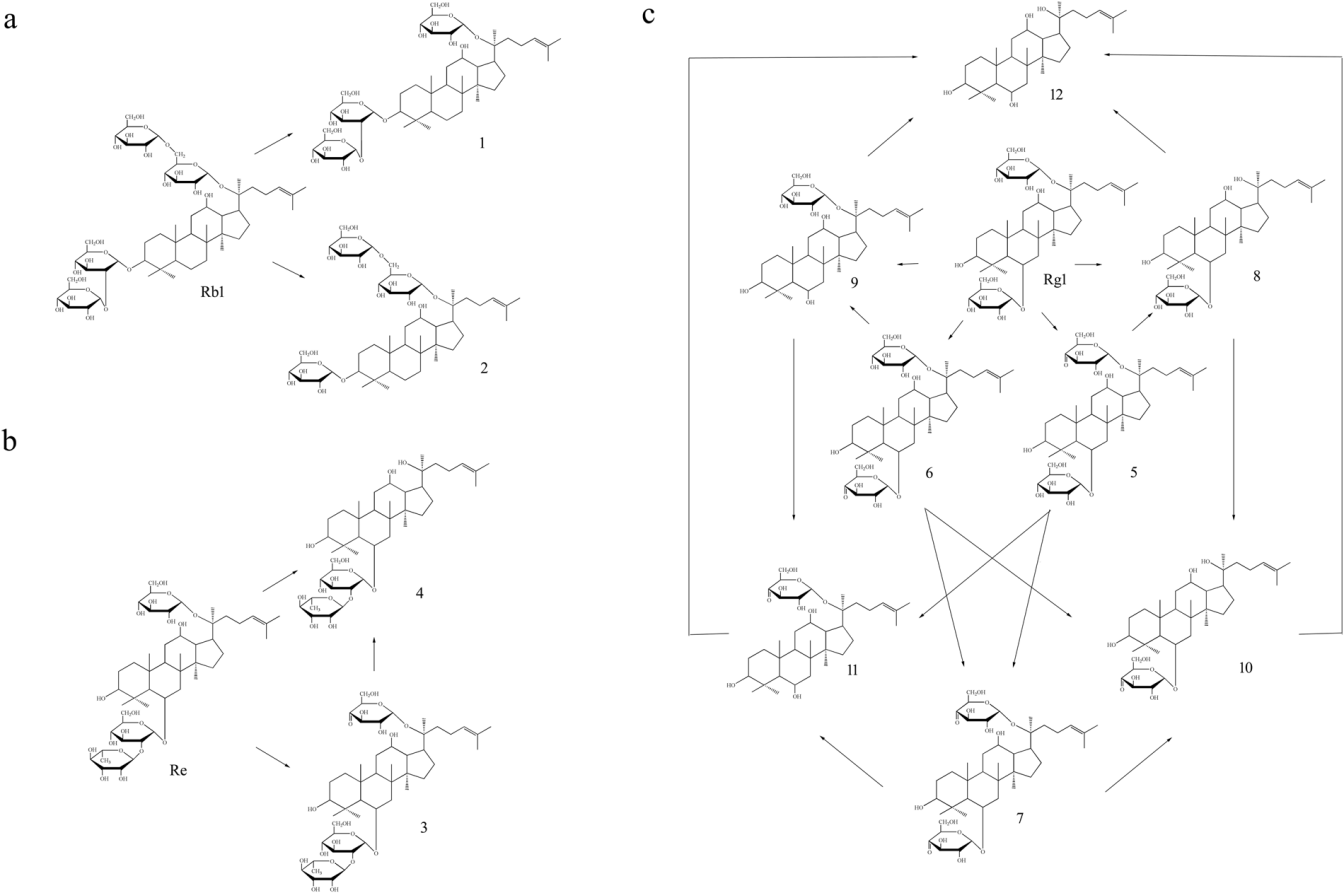
Biotransformation pathways of ginsenoside Rb1 (**a**), Re (**b**), and Rg1 (**c**) during biotransformation process by using *Cellulosimicrobium* sp. TH-20.

The microbial transformation pathway of Rb1 was proposed in Fig. 2a. Rb1 was transformed by using *Cellulosimicrobium* sp. TH-20 via deglycosylation pathways: Rb1 → Rd/GypXVII. Rd was formed by cleavage of β-D-glucopyranose linkage of 20-O-β-D-glucospyranosyl-(1 → 6)-β-D-glucopyranose from Rb1. And GypXVII was created by hydrolysis of β-D-glucopyranose linkage of 3-O-β-D-glucospyranosyl-(1 → 2)-β-D-glucopyranose from Rb1.

As shown in Fig. 2b, Re was transformed by using *Cellulosimicrobium* sp. TH-20 via deglycosylation and dehydrogenation. The possible pathways were as follows: Re → Rg2 or Re → 20-C-4-carbonyl-glycuronic Re → Rg2. 20-C-4-carbonyl-glycuronic Re was an intermediate in the bioconversion from Re to Rg2. Dehydrogenation on glucose moiety at the C20 position of Re led to the formation of 20-C-4-carbonyl-glycuronic Re. And, hydrolysis of glucose and dehydroglucose moieties attached to the C20 position of Re and 20-C-4-carbonyl-glycuronic Re, respectively, resulted in conversion to Rg2.

The microbial transformation pathway of Rg1 by using *Cellulosimicrobium* sp. TH-20 was proposed in Fig. 2c. In total, 8 metabolites including Rh1, F1, PPT, 20-C-4-carbonyl-glycuronic Rg1, 6-C-4-carbonyl-glycuronic Rg1, 6,20-C-4-carbonyl-glycuronic Rg1, 20-C-4-carbonyl-glycuronic F1 and 6-C-4-carbonyl-glycuronic Rh1 were transformed from ginsenoside Rg1 via deglycosylation and dehydrogenation. And PPT was the final biotransformation product. Dehydrogenation of sugar moieties at C6 or/and C20 position of Rg1 led to the formation of 20-C-4-carbonyl-glycuronic Rg1, 6-C-4-carbonyl-glycuronic Rg1, and 6,20-C-4-carbonyl-glycuronic Rg1. Similarly, 20-C-4-carbonyl-glycuronic F1 and 6-C-4-carbonyl-glycuronic Rh1 were metabolized via dehydrogenation on sugar moieties at C20 position of F1 and C6 position of Rh1, respectively. Moreover, deglycosylation on two types of sugar moieties, including glucose and dehydroglucose moieties, with linkage position at C6 or C20 of aglycon occurred in the biotransformation process. Hydrolysis of the glucose moieties attached to the C6 position of aglycon led to the production of F1, 20-C-4-carbonyl-glycuronic F1 and PPT from Rg1, 20-C-4-carbonyl-glycuronic Rg1 and Rh1, respectively. Cleavage of the glucose moiety at C20 position of aglycon resulted in the formation of Rh1, 6-C-4-carbonyl-glycuronic Rh1 and PPT from Rg1, 6-C-4-carbonyl-glycuronic Rg1 and F1, respectively. In addition, dehydroglucose moieties at the C6 or C20 position of aglycon can be hydrolyzed. The F1, 20-C-4-carbonyl-glycuronic F1 and PPT could be produced by hydrolysis of the dehydroglucose moiety at C6 position of 6-C-4-carbonyl-glycuronic Rg1, 6,20-C-4-carbonyl-glycuronic Rg1 and 6-C-4-carbonyl-glycuronic Rh1, respectively. Moreover, the Rh1, 6-C-4-carbonyl-glycuronic Rh1 and PPT could be created by cleavage of dehydroglucose moiety at C20 position of 20-C-4-carbonyl-glycuronic Rg1, 6, 20-C-4-carbonyl-glycuronic Rg1 and 20-C-4-carbonyl-glycuronic F1, respectively. Hence, based on the analysis of the biotransformation pathway of ginsenoside Rg1, it is concluded that PPT could be converted from Rg1 via 12 possible pathways.

Our study revealed multiple transformation pathways of ginsenosides by using *Cellulosimicrobium* sp. TH-20, including deglycosylation and dehydration on PPD-type and PPT-type ginsenosides. Among these biotransformation pathways, 11 were first identified and unique compared with previous reports on the microbial transformation of ginsenosides. The characterization of the biotransformation process would be helpful for understanding the transformation mechanism of ginsenosides by using *Cellulosimicrobium* sp. TH-20.

### Quantative analysis of the biotransformation process by *Cellulosimicrobium* sp. TH-20 using HPLC

**Figure 3:**
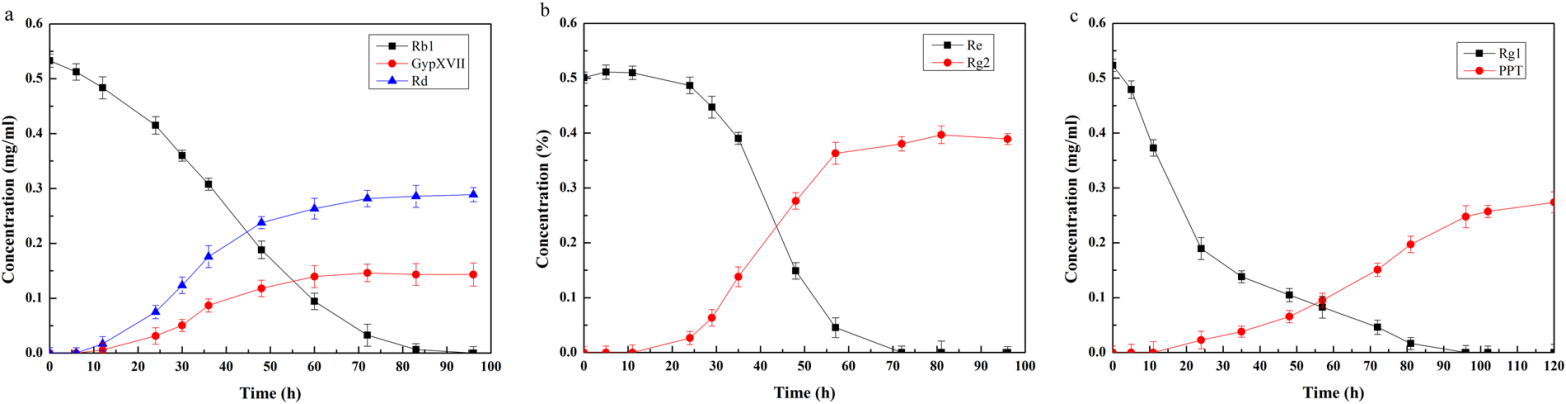
Time courses of ginsenoside Rb1 (**a**), Re (**b**), and Rg1 (**c**) during biotransformation process by using *Cellulosimicrobium* sp. TH-20.

To date, efficient methods are limited for producing ginsenosides, especially for rare ginsenosides. The time courses of the biotransformation of ginsenoside Rb1, Re and Rg1 using *Cellulosimicrobium* sp. TH-20 were investigated through HPLC analysis (Fig. 3). Ginsenoside Rb1 and Rg1 were exhausted at 96 h, while Re was metabolized thoroughly at 36 h. In total, 16 mg of Rd and 10 mg of GypXVII can be produced from 30 mg of Rb1, with yields of 61% and 38%, respectively. Similarly, 24 mg of Rg2 can be produced from 30 mg of Re with a yield of 96%. In addition, 16 mg of PPT can be generated from 30 mg of Rg1 with a yield of 89%. Hence, the production of ginsenosides Rd, Rg2, PPT and GypXVII by *Cellulosimicrobium* sp. TH-20 makes the pharmaceutical study possible.

### Effects of ginsenosides on LPS-stimulated RAW 264.7

The effect of ginsenosides on cell viability was evaluated using the CCK-8 assay (Supplementary Fig. S10). Following 24-h treatment, ginsenosides at concentrations ranging from 45 to 180 µM had no effect on RAW264.7 cell viability. Hence, these ginsenoside concentrations were considered suitable for further assays.

**Figure 4:**
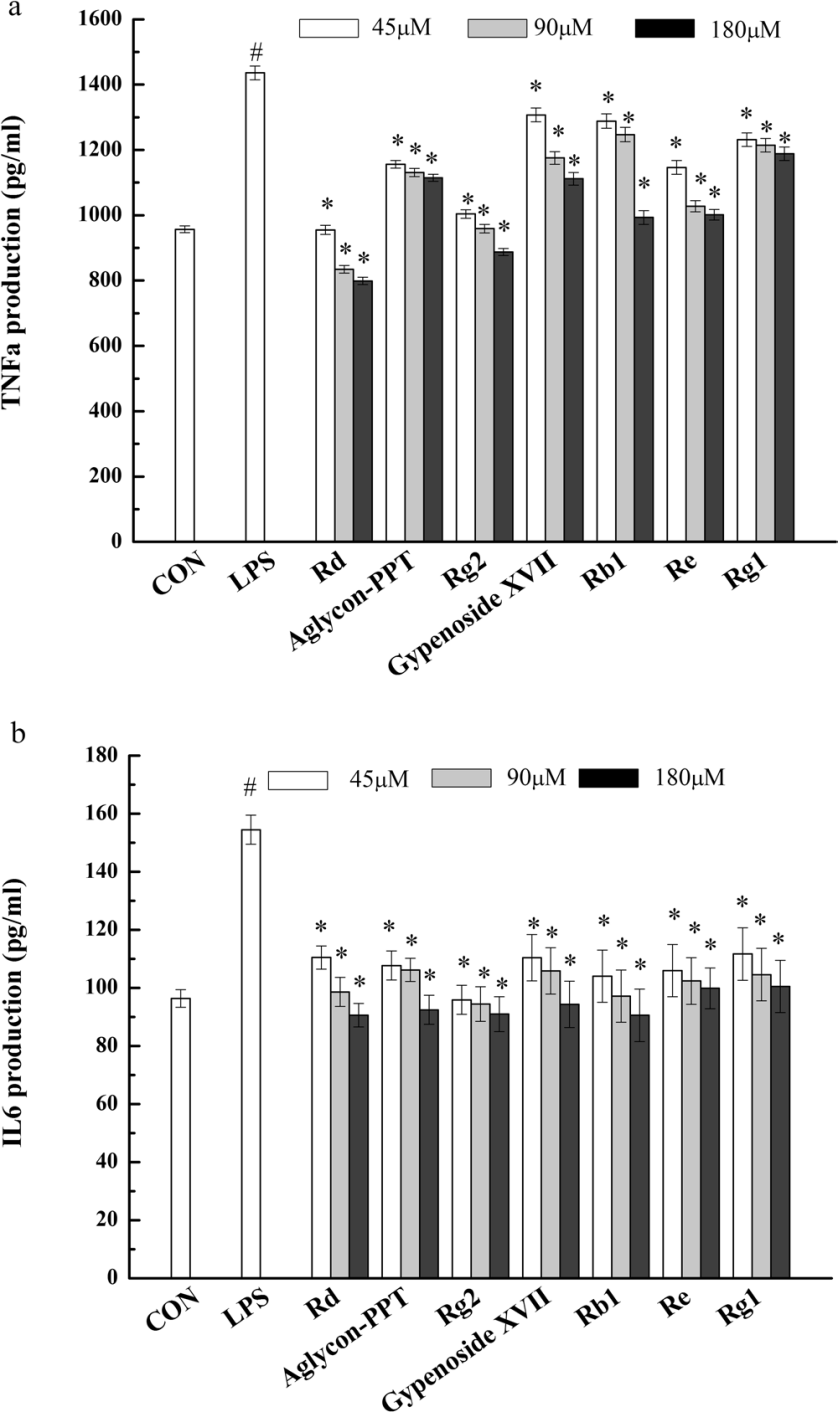
Effects of ginsenosides on protein productions of pro-inflammatory cytokines TNF-α (**a**) and IL-6 (**b**) in LPS-stimulated RAW 264.7 cells. The data are means ± SD (n = 3). ^#^p < 0.05 vs. the LPS-free control; *p < 0.05 vs. the LPS treated group.

Pro-inflammatory cytokines play a key role in the process of inflammation and associated diseases^23, 24^. To investigate the anti-inflammatory activity of ginsenosides at the cell level, we examined the effects of ginsenosides, including major ginsenosides (Rb1, Re, Rg1) and the transformed ginsenosides (Rd, GypXVII, Rg2 and PPT), on the production of pro-inflammatory cytokines TNF-α and IL-6 in LPS-stimulated RAW 264.7 cells. As noted in Fig. 4a and b, the amount of TNF-α and IL-6 in cell culture medium was markedly increased (1435 pg/ml and 155 pg/ml) in the model group subjected to 24 h LPS (1 μg/mL) treatment versus the control group that did not receive LPS stimulation. Compared with the model, treatment with all of these seven ginsenosides significantly inhibited LPS-induced TNF-α and IL-6 production in a dose-dependent manner. Moreover, the transformed ginsenosides (Rd, GypXVII, PPT and Rg2) shown in Fig. 4a dramatically attenuate the production of TNF-α compared with the major ginsenosides, indicating better efficacy compared with major ginsenosides. However, IL-6 production inhibition was not significantly different between all ginsenosides groups (Fig. 4b). These results indicated that the transformed ginsenosides have greater anti-inflammatory effects than the major ginsenosides in LPS-stimulated RAW 264.7 cells.

### Effects of ginsenosides on mouse ear swelling response

**Figure 5:**
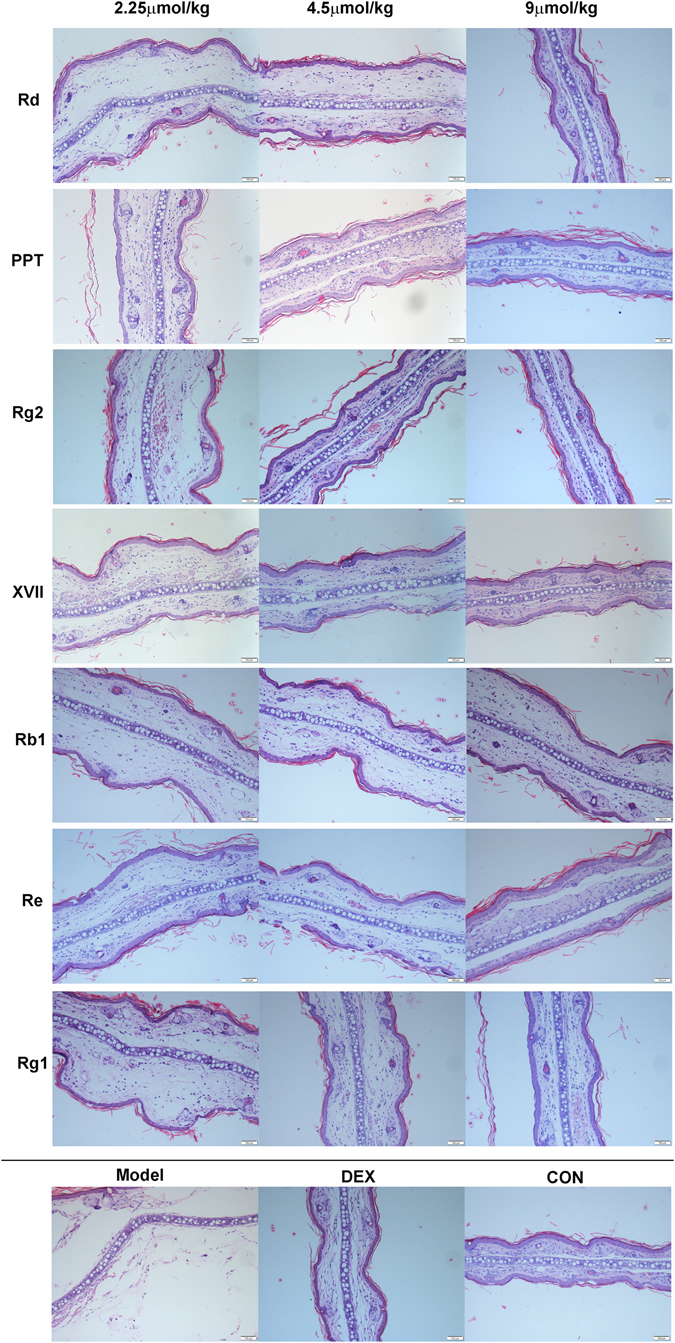
Histopathology analysis of the inhibitory effect of ginsenosides on xylene-induced inflammatory response in mouse ear swelling. Scale bars, 100 μm. Magnification, 200×.

**Figure 6:**
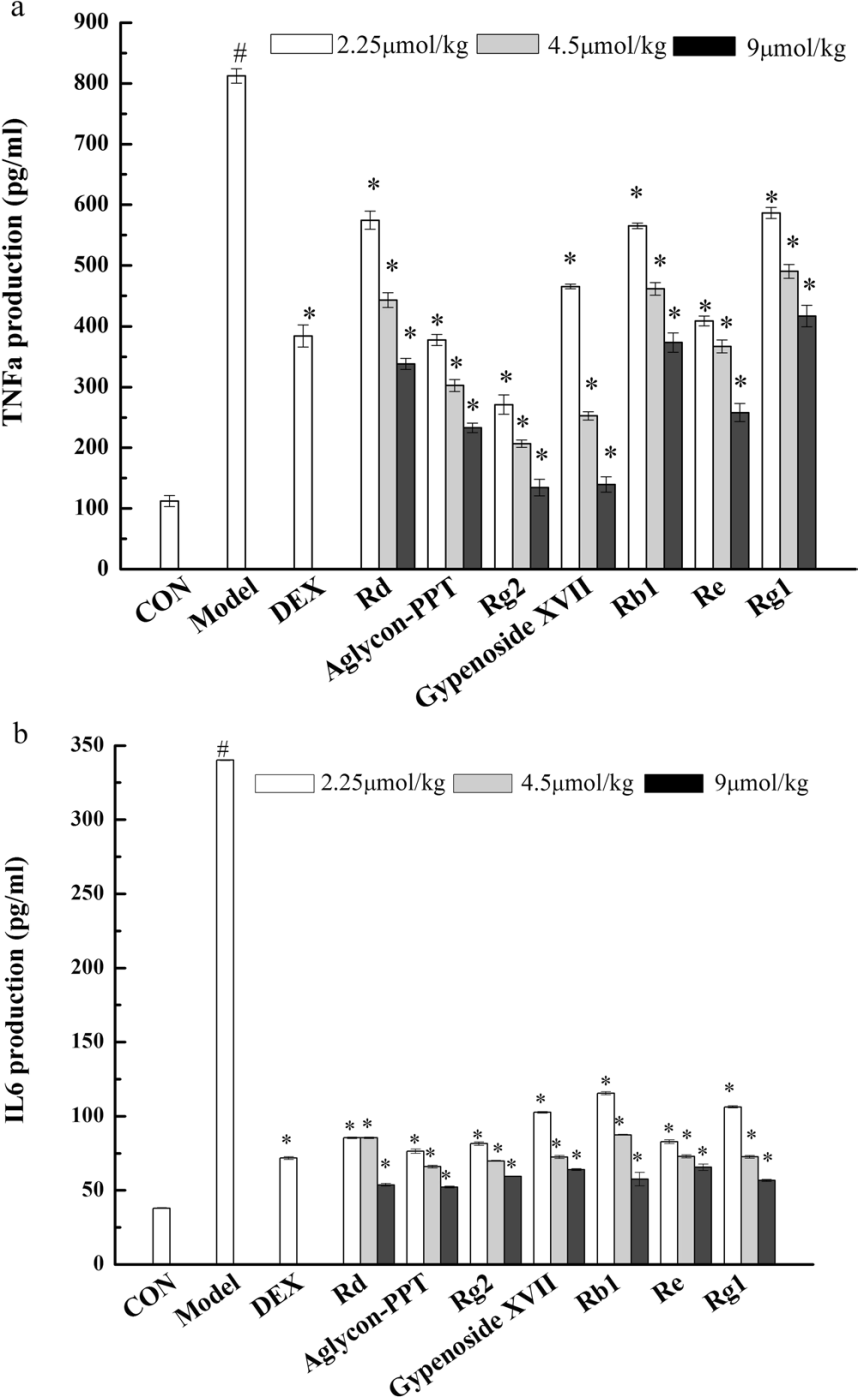
Effects of ginsenosides on protein productions of pro-inflammatory cytokines TNF-α (**a**) and IL-6 (**b**) in the xylene-induced ear swelling model. The data are means ± SD (n = 3). ^#^p < 0.01 vs. the control group; *p < 0.001 vs. the model group.

Mouse ear swelling assays were conducted^25^ to evaluate the anti-inflammatory activity of ginsenosides including rare ginsenosides Rd, GypXVII, Rg2 and PPT, which are the final products by microbial transformation with *Cellulosimicrobium* sp. TH-20, and the precursors. In vehicle-treated controls, the right ears exhibited obvious inflammatory symptoms including redness and swelling. However, mice pretreated with these ginsenosides or the positive drug control dexamethasone exhibited significantly reduced swelling. All these ginsenosides at concentration ranges of 2.25- to 9 µmol/kg exhibited significant inhibitory effects on xylene-induced ear swelling. The biotransformed products Rd, GypXVII, Rg2 and PPT exhibit higher anti-inflammatory activity on acute xylene-induced inflammation compared to the ginsenosides Rb1, Re, and Rg1, and the maximum inhibition rate was as high as 85% (Rg2, 9µmol/kg) (Supplementary Fig. S11). We also observed the differences in the thickness of the histological structures of the punch ears. As noted in Fig. 5, the model group showed the maximum thickness compared to the groups treated with ginsenosides. In the ginsenosides-treated groups, the tissues exhibited a dose-dependent reduction in thickness. In the cases of groups that treated with high concentrations of Rd, PPT, XVII, and Rg2, the tissues return to normal structure comparable with the control group. Furthermore, the ginsenosides also caused changes in the concentration of pro-inflammatory cytokines (TNF-α, and IL-6) in this model of acute inflammation in a dose-dependent manner. As shown in Fig. 6a and b, the biotransformed products showed higher inhibitiory activities than the precursors. For TNF-α, the maximum inhibition rate was 72% (Rg2, 9 μmol/kg), and for IL-6 the maxium inhibition rate was 85% (PPT, 9 μmol/kg). Hence, the four products showed improved anti-inflammatory activities by using lipopolysaccharide (LPS)-induced murine RAW 264.7 macrophages and the xylene-induced acute inflammatory model of mouse ear edema.

## Materials and Methods

### Materials

Ginseng root extract was purchased from ZeLang Healthtech Co. Ltd., China. Reference ginsenosides, including ginsenoside Rb1, Re, Rg1, Rd, Gyp XVII, Rg2, F1, Rh1 and PPT were purchased from Jilin University (Changchun, China). Acetonitrile and methanol of HPLC grade were obtained from Fisher Scientific (Waltham, MA). HPLC grade formic acid (96%) was obtained from Tedia (Fairfield, OH). Other reagents were of analytical purity.

The RAW264.7 cell line was purchased from the Type Culture Collection of the Chinese Academy of Sciences (Shanghai, China). Dulbecco’s modified Eagle’s medium (DMEM) was obtained from Gibco (Carlsbad, USA). Lipopolysaccharides (LPS) was purchased from Sigma (Missouri, USA). A Cell Counting Kit-8 (CCK-8) and enzyme-linked immunosorbent assay (ELISA) kit were obtained from Bestbio (Shanghai, China) and R&D (Minneapolis, USA).

### Screening of ginsenoside-hydrolyzing bacteria

To screen effective microorganisms that can transform ginsenosides, the ginseng soil samples were collected and cultured on ginseng agar medium plates. Microorganisms that utilize ginsenosides as an exclusive source of carbon were selected for further study. Soil samples from 5-to 10 cm below ground were collected from ginseng growing field (Fusong, China). After 10^−3^- to 10^−7^ dilution with sterile water, 1 mL of soil samples was spread on the ginseng agar medium plates[0.3% (w/v) NaNO_3_, 0.1% (w/v) K_2_HPO_3_•3H_2_O, 0.05% (w/v) MgSO_4_, 0.01% (w/v) FeSO_4_•7H_2_O) and 1.0% (w/v) ginseng root extract]. The ginsenoside-hydrolyzing ability of the pure cultures was screened by TCL and HPLC analysis.

### Identification of strain TH-20

The isolated strain TH-20 was identified through morphological observation, biochemical characteristics and phylogenetic analysis. A suspension of the strain was treated with glutaraldehyde, tannic acid and gradient ethanol and then covered by gold to be observed using scanning electron microscope (XL30 ESEM FEG, FEI Co., The Netherlands). Tests of biochemical characteristics were performed according to the conventional bacteria identification manual. The 16 S rDNA gene of *Cellulosimicrobium* sp. TH-20 was sequenced. The 16 S rDNA gene sequences of related taxa were obtained from GenBank (National Center for Biotechnology Information; Bethesda, MD, USA), and a phylogenetic tree was constructed via the neighbor-joining method using the MEGA 3.1 program^26^. A bootstrap analysis with 1,000 replicates was conducted to obtain confidence levels for the branches.

### Biotransformation of ginsenosides Rb1, Re and Rg1 by *Cellulosimicrobium* sp. TH-20

The biotransformation procedure was performed using biotransformation medium (0.3% (w/v) NaNO_3_, 0.1% (w/v) K_2_HPO_3_•3H_2_O, 0.05% (w/v) MgSO_4_, 0.01% (w/v) FeSO_4_•7H_2_O) with 125 mg l^−1^ Rb1, Re or Rg1 as the carbon source in a shaking incubator (170 rpm) at 30 °C for 5 days. All medium was sterilized at 121 °C for 20 min and the initial pH was 7.0. Samples were withdrawn at regular intervals during fermentation. An equal volume of n-butanol saturated with water was added to stop the reaction. After centrifugation (4 °C, 10,000 rpm), the reactant present in the n-butanol fraction was evaporated in a water bath at 60 °C. Samples were redissolved in methanol and diluted to 1.0 mL for further analysis.

### Analytic methods

#### TLC analysis

TLC analysis was carried out using a Silica Gel 60 plates and a solvent system of CHCl_3_-CH_3_OH-H_2_O (13:7:2 v/v/v) as the developing solvent. The spots on the TLC plates were detected by spraying 10% (v/v) H_2_SO_4_ (in ethanol) followed by heating at 110 °C for 10 min.

#### HPLC analysis

Chromatographic separation was performed on an Agilent 1200 series HPLC system (Agilent Technologies, USA) equipped with a vacuum degasser, a binary pump, an autosampler, a thermostatically controlled column apartment and connected to a diode array detector (DAD). Samples were loaded onto a C18 reverse-phase chromatographic column (5 cm × 3.0 mm, 2.7 μm; Supelco. USA). The mobile phase consisted of water (A) and acetonitrile (B). The gradient elution was programmed as follows: 0–2.5 min, 19% (B); 2.5–5 min, 19–30% (B); 5–11 min, 30–33% (B); 11–20 min, 33–45% (B); 20–25 min, 45–65% (B). The detection wavelength was 203 nm. The column temperature was 35 °C, the flow rate was 0.2 mL/min and the injection volume was 5 μL.

#### RRLC-Q-TOF MS analysis

LC analysis was performed using an Agilent 1200 RRLC system equipped with a vacuum degasser, a quaternary pump, an autosampler, and a thermostatically controlled column apartment. The separation conditions were the same as that used in the HPLC analysis. The RRLC system was connected to an Agilent 6520 Q-TOF mass spectrometer equipped with an electrospray interface. The scan range for MS acquisition was from 100 to 2200 m/z in negative ionization mode. The conditions of the electrospray ionization (ESI) source were as follows: drying gas, N2; flow rate, 4.0 L/min; drying gas temperature, 350 °C; nebulizer, 30 psig; capillary voltage, 3500 V; fragmentor, 350 V; cone voltages, 65 V. Data analysis was performed by Mass Hunter Qualitative (MHQ) software, version B.03.01 (Agilent Technologies, Santa Clara, CA).

#### UHPLC-Q-Exactive Orbitrap HRMS analysis

UHPLC-Q-Exactive Orbitrap HRMS analysis was performed on the Thermo Fisher Scientific Dionex Ultimate 3000 HPLC system (UHPLC) equipped with quaternary analytical pump, solvent rack SR-3000, w/o degasser, well plate sampler WPS-3000SL analytical and TCC-3000RS. The C18 reverse-phase chromatographic column (10 cm × 2.2 mm, 1.7 μm; Thermo Fisher Scientific, USA) was used for chromatographic separation of target compounds. Formic acid (0.1%, v/v) and acetonitrile were used as the mobile phases A and B, respectively. The gradient elution was programmed as follows: 0–6.7 min, 15% (B); 6.7–13.3 min, 15–19% (B); 13.3–17.3 min, 19–25% (B); 17.3–21.3 min, 25–36% (B); 21.3–26.7 min, 36–45% (B); 26.7–33.3 min, 45–65% (B); 33.3–37.3 min, 65–80% (B); 37.3–46.7 min, 80–100% (B); 46.7–53.3 min, 100% (B). The column temperature was maintained at 35 °C, the flow rate was 0.2 mL/min, and the injection volume was 5 μL.

Detection of compounds was performed using a Q Exactive mass spectrometer equipped with electrospray ionization (ESI) source. The scan range for MS/MS acquisition was from 50 to 875 m/z in negative ionization mode. The conditions of the electrospray ionization (ESI) source were as follows: Spray voltage, 3.5 kV; Sheath gas, N2; pressure, 140KPa; Aux gas, N_2_; flow rate 1.7 L/min; capillary temperature, 320 °C; probe heater temperature, 30 °C. The data were evaluated by the Quan/Qual Browser Xcalibur 2.3 software (Thermo Fisher Scientific, Waltham, MA).

### Assessment of anti-inflammatory activity *in vivo* and *in vitro*

#### Cell culture and treatment

RAW264.7 mouse macrophage cells were cultured in DMEM medium supplemented with 10% FBS, glutamine, and antibiotics at 37 °C under 5% CO_2_. Cells at 80–90% confluency were centrifuged at 120 × g at 4 °C for 10 min. The cell concentration was adjusted to (2 × 10^6^) cells/ml, and the cell viability was consistently greater than 90%. A 50-μL cell suspension was seeded into a tissue culture grade 96-well plate (4 × 10^5^ cells/well) and incubated for 2 h at 37 °C and 5% CO_2_ for cell attachment. Then, cells were stimulated by using 1 μg/mL of LPS with or without the presence of ginsenosides tested at the final volume of100 μL/well. Cells were further incubated at 37 °C and 5% CO_2_ for 24 h for use.

#### Cell viability

Cell viability was measured using a CCK-8 assay. Briefly, 4 × 10^4^ cells/well RAW264.7 cells were plated in 96-well plates, incubated for 2 h, and then treated with different concentrations (10, 20 and 40 mg/ml) of ginsenosides (Rd, GypXVII, PPT and Rg2) for 24 h. An equal volume of medium was used as blank control. After treatment, cells were incubated with 10 μl of CCK-8 for an additional 4 h in the dark. The absorbance at 450 nm was recorded using a PR 4100 microplate reader (Bio-Rad Inc., Hercules, CA, USA).

#### Analysis of cytokines using ELISA assay

TNF-α and IL-6 concentrations in the cell culture supernatant or the serum were detected using an ELISA kit according to the manufacturer’s instruction, and the results were expressed in pg/mL of protein. All of the analyses were performed in triplicate.

#### Xylene-induced ear swelling and hisological analysis

All experiments involving the use of animals in this study were approved by College of Basic Medical Sciences, Jilin University and performed in accordance with the animal welfare guidelines outlined in the Guide for the Care and Use of Laboratory Animals. Fifty mice were divided randomly into five groups (A–E). Groups B-D were administered ginsenosides through tail vein injection at one of three different doses (2.25 µmol/kg, 4.5 µmol/kg, or 9 µmol/kg) for six days. The mice in the remaining groups were used as either vehicle controls (A) or positive controls (E) and were administered the same volume of physiological saline or dexamethasone (15 mg/kg), respectively. One hour after the sixth day of administration, animals received a 20-μL smear of xylene on both the anterior and posterior surfaces of the right ear lobe. Two hours later, take blood from the eyeballs of mice and euthanized the mice. The serum was collected by centrifugation at 3000 rpm, 10 min. 9-mm sections were obtained from both ears with a cork borer. The tissue was subsequently weighed. The degree of ear swelling was calculated according to the following formula: Ear swelling inhibition rate (%) = (A − B)/A * 100, where A and B denote ear swelling of the negative group and ear swelling of the drug groups, respectively. For histological analysis, ear biopsy samples were fixed with 4% paraformaldehyde, embedded in paraffin, and sectioned at a thickness of 9 mm. Sliced sections were stained with hematoxylin and eosin (H&E).

#### Statistical analysis

Statistical analyses were performed with SPSS 13.0 software (SPSS Inc., USA). The data were analyzed by one way ANOVA followed by Student’s two-tailed t test for comparison between two groups, and the data are presented as the mean ± SD. P < 0.05 was statistically significant difference.

## Electronic supplementary material

supplementary information
