# Supplementary material for: Microbial transformation of ginsenoside Rb1, Re and Rg1 and its contribution to the improved anti-inflammatory activity of ginseng
Source: Sci Rep. 2017 Mar 10;7:138. doi: 10.1038/s41598-017-00262-0 (PMC5428039; doi:10.1038/s41598-017-00262-0)
Supplement: Supplementary file 1 — supplementary information [file 41598_2017_262_MOESM1_ESM.doc]

Supplementary Information

Microbial transformation of ginsenoside Rb1, Re and Rg1 and its contribution to the improved anti-inflammatory activity of ginseng

Shanshan Yu^1,2^*, Xiaoli Zhou^3^, Fan Li^4^, Chunchun Xu^2^, Fei Zheng^2^, Jing Li^2^, Huanxi Zhao^2^, Yulin Dai^2^, Shuying Liu ^2^ and Yan Feng^1^

*1 State Key Laboratory of Microbial Metabolism, School of Life Sciences and Biotechnology, Shanghai Jiao Tong University, Shanghai 200240, China.*

*2 Jilin Ginseng Academy, Changchun University of Chinese Medicine, Changchun 130117, China.*

*3College of Basic Medical Sciences, Jilin University, Changchun 130021, Jilin, China.*

*4 School of Life Sciences, Northeast Normal University, Changchun 130024, China.* Correspondence and requests for materials should be addressed to Y.F. ([yfeng2009@sjtu.edu.cn](mailto:yfeng2009@sjtu.edu.cn)) or S.Y.L. ([syliu19@yahoo.com.cn](mailto:syliu19@yahoo.com.cn))

**Screening of ginsenoside-transforming bacteria**

In order to screen appropriate microbial strains for ginsenosides production, soil samples from ginseng field were collected. More than 20 microorganisms were isolated on ginseng agar medium plates. After major ginsenosides including Rb1, Rb2, Rc, and Rg1 were fed to these isolates, the transformation products were determined through thin layer chromatography (TLC) analysis. Ten isolates were confirmed to exhibit ginsenoside-transforming activities (Supplementary Fig. S1).Strain TH-1, TH-5, TH-10, and TH-17 exhibited Rb1-transforming activities. In addition, TH-6 TH-12, andTH-15 exhibited PPD-type-transforming activities. Similarly, strain TH-4 and TH-13 exhibited PPT-type-transforming activities.Compared to other isolates, strain TH-20 exhibited unique and effective activities to transform both PPD and PPT-type ginsenosides.

**Structure identification ofintermediate metabolites 3, 5, 6, 7, 10 and 11**

Structures of intermediates 3, 5, 6, 7, 10 and 11 were determined via RRLC-Q-TOF MS/MS and UHPLC-Q-Exactive Orbitrap HRMS analysis (Supplementary Fig. S4-9). The accurate mass, the isotopic ratio pattern and the characteristic fragment ions obtained from RRLC-Q-TOF MS/MS and UHPLC-Q-Exactive Orbitrap HRMS analyses are helpful for identifying compounds. The error for all molecular ions and fragment ions was less than 10 ppm. Given that 0.1% formic acid solution was employed as the mobile phase, each ginsenoside exhibited the deprotonated ion [M-H]^−^ and adduct ion [M+HCOOH]^−^ in negative-ion mode, providing information regarding the molecular mass.

As noted in Supplementary Fig. S4a, metabolite 3 exhibited a [M+HCOO]^−^ ion at *m/z* 989.5582 in full scan spectrum. The MS/MS fragmentation exhibited an ion at *m/z* 943.5477 and included Y_0α_ (*m/z* 783.5162), Y_1β_ (*m/z* 637.4632), Y’_0β_ (*m/z* 475.4030), B_1α_ (*m/z* 161.0528) and ^2,5^A_1α_ (*m/z* 101.0328). The characteristic ion at *m/z* 475.4030 was observed, which is typical for PPT-type ginsenosides. The [M+HCOOH]^–^ ion at *m/z* 989.5582 indicates a -2 Da mass difference compared with the [M+HCOOH]^–^ ion of Re ion at *m/z* 991.5477, indicating that dehydrogenation is occurring in Re. The ion at *m/z* 783.5291 (Y_0α_) was produced by the lose of a dehydroglucoside (160 Da) from the ion at *m/z* 943.5477, indicating that the glucose moiety at C20 position of Re was dehydrogenated.

UHPLC-Q-Exactive Orbitrap HRMS was further applied to identify the position of the aldehyde group in the dehydroglucoside. Dehydrogenation of the glucose moiety at the C20 position of Re will result in three possible products, including 2-carbonyl-glucose aldehyde, 3-carbonyl-glucose aldehyde and 4-carbonyl-glucose aldehyde. The fragment ions of these three possible products were summarized. The fragment ions of 2-carbonyl-glucose aldehyde were ^0,4^A_1α_(*m/z* 59), ^0,3^A_1α_(*m/z* 89), ^0,2^A_1α_(*m/z* 119), ^0,4^X_0α_(*m/z* 99), ^0,3^X_0α_(*m/z* 69) and ^0,2^X_0α_(*m/z* 39). The fragment ions of 3-carbonyl-glucose aldehyde were ^0,4^A_1α_(*m/z* 59), ^0,3^A_1α_(*m/z* 89), ^0,2^A_1α_(*m/z* 117), ^0,4^X_0α_(*m/z* 99), ^0,3^X_0α_(*m/z* 69) and ^0,2^X_0α_(*m/z*41). The fragment ions of 4-carbonyl-glucose aldehyde were ^0,4^A_1α_(*m/z* 59), ^0,3^A_1α_(*m/z* 87), ^0,2^A_1α_(*m/z* 117), ^0,4^X_0α_(*m/z* 99), ^0,3^X_0α_(*m/z* 71) and ^0,2^X_0α_(*m/z* 41). Hence, ions at *m/z* 87 and *m/z* 71 were characteristic fragment ions for 4-carbonyl-glucose aldehyde. As noted in Supplementary Fig. S4b, in MS/MS spectrum of the [M+HCOOH]^−^ ion at *m/z* 989.5582, ions, including ^0,3^A_1α_ at *m/z* 87 and ^0,3^X_0α_at *m/z* 71, were identified. Thus, metabolite 3 was identified as 20-C-4-carbonyl-glycuronic Re, which is a novel ginsenoside that differs from other previously described ginsenosides.In the analysis of metabolites 5, 6, 7, 10 and 11, UHPLC-Q-Exactive Orbitrap HRMS was also used to identify the position of the aldehyde group in the dehydroglucoside. Collectively, the mass spectrum data suggested that the aldehyde group was formed at C4 position of the glucose moiety.

As noted in Supplementary Fig. S5 and S6, metabolites 5 and 6 both exhibited a [M+HCOO]^−^ ion at *m/z* 843 that was -2 Da mass different with the [M+HCOO]^–^ ion of Rg1 at *m/z* 845.4898. 5 and 6 were isomers. Rg1 possessed two glucose moieties with one at C6 position and another at C20 position. The ions at *m/z* 637.4374 and 637.4406 were produced by the neutral loss of 160 Da and HCOOH (46Da) from [M+HCOO]^−^ ion at *m/z* 843.4865 and 843.4894, respectively, suggesting that one of the two sugar moieties was dehydrogenated in Rg1. Thus, 5 and 6 were identifiedto be 6-C-4-carbonyl-glycuronic Rg1 and 20-C-4-carbonyl-glycuronic Rg1, respectively. The peak order of 6-C-4-carbonyl-glycuronic Rg1 and 20-C-4-carbonyl-glycuronic Rg1 refers to the peak order of Rh1 and F1. As noted in Fig. 1C, the retention time decreased with the increasing polarity of ginsenoside. Given that the structural difference between Rh1 and F1 lies in the glucose moiety position, it is inferred that the glucose moiety at C6 position exhibits stronger polarity than C20 position. Hence, 5 was deduced as 20-C-4-carbonyl-glycuronic Rg1, and 6 was deduced as 6-C-4-carbonyl-glycuronic Rg1. As noted in Supplementary Fig. S7, 7 exhibited a [M-H]^−^ ion at *m/z* 795.4767, which exhibited a -4 Da mass difference with the [M-H]^−^ ion of Rg1 at *m/z* 800.4922. Thus, 7 was 6, 20-C-4-carbonyl-glycuronic Rg1 based on mass spectrum data. Based on similar analyses, 10 with a -2 Da mass difference with the [M-H]^−^ ion of Rh1 at *m/z* 637.4316 was identified to be 20-C-4-carbonyl-glycuronic Rh1, whereas 11 with a -2 Da mass difference with the [M-H]^−^ ion of F1 at *m/z* 637.4316 was identified to be 6-C-4-carbonyl-glycuronic F1 (Supplementary Fig. S8 and S9).


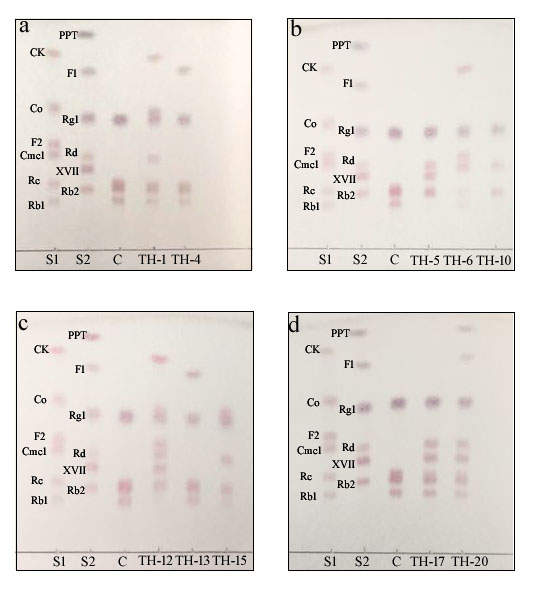


**Figure S1**. TLCanalysis of ginsenoside-transforming activities of microorganisms isolated from soil samples on which ginseng grown.C:major ginsenosides including Rb1, Rb2, Rc, and Rg1; S1 and S2: ginsenosides standards.


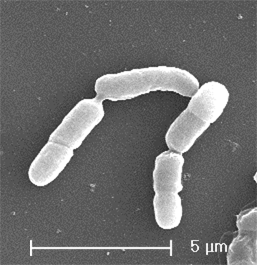


**Figure S2.** Morphology of strain TH-20 with 4864× magnification .


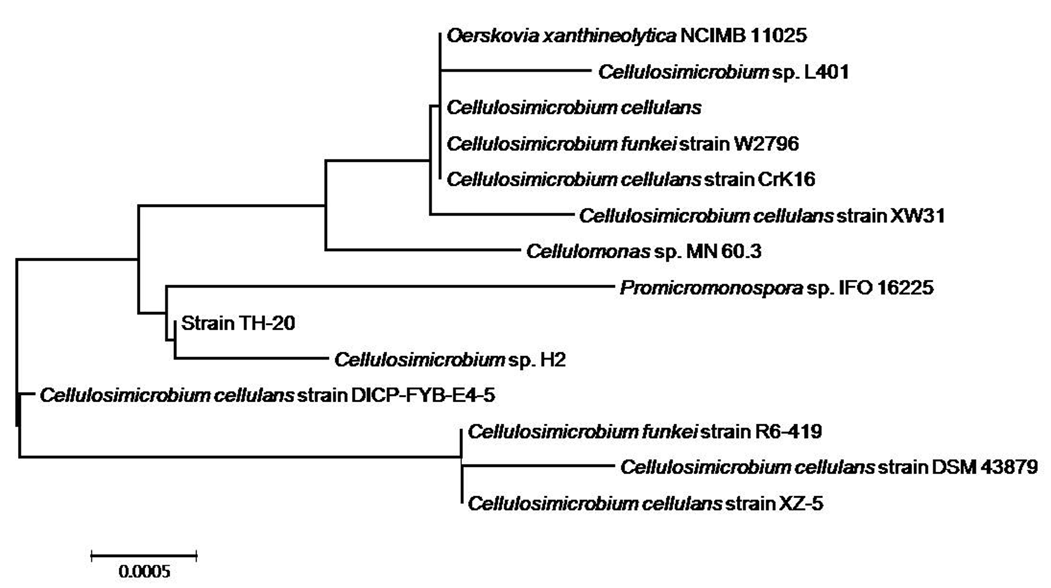


**Figure S3**. Phylogenetic tree based on the 16s rDNA sequence of strain TH-20 and related microorganisms.


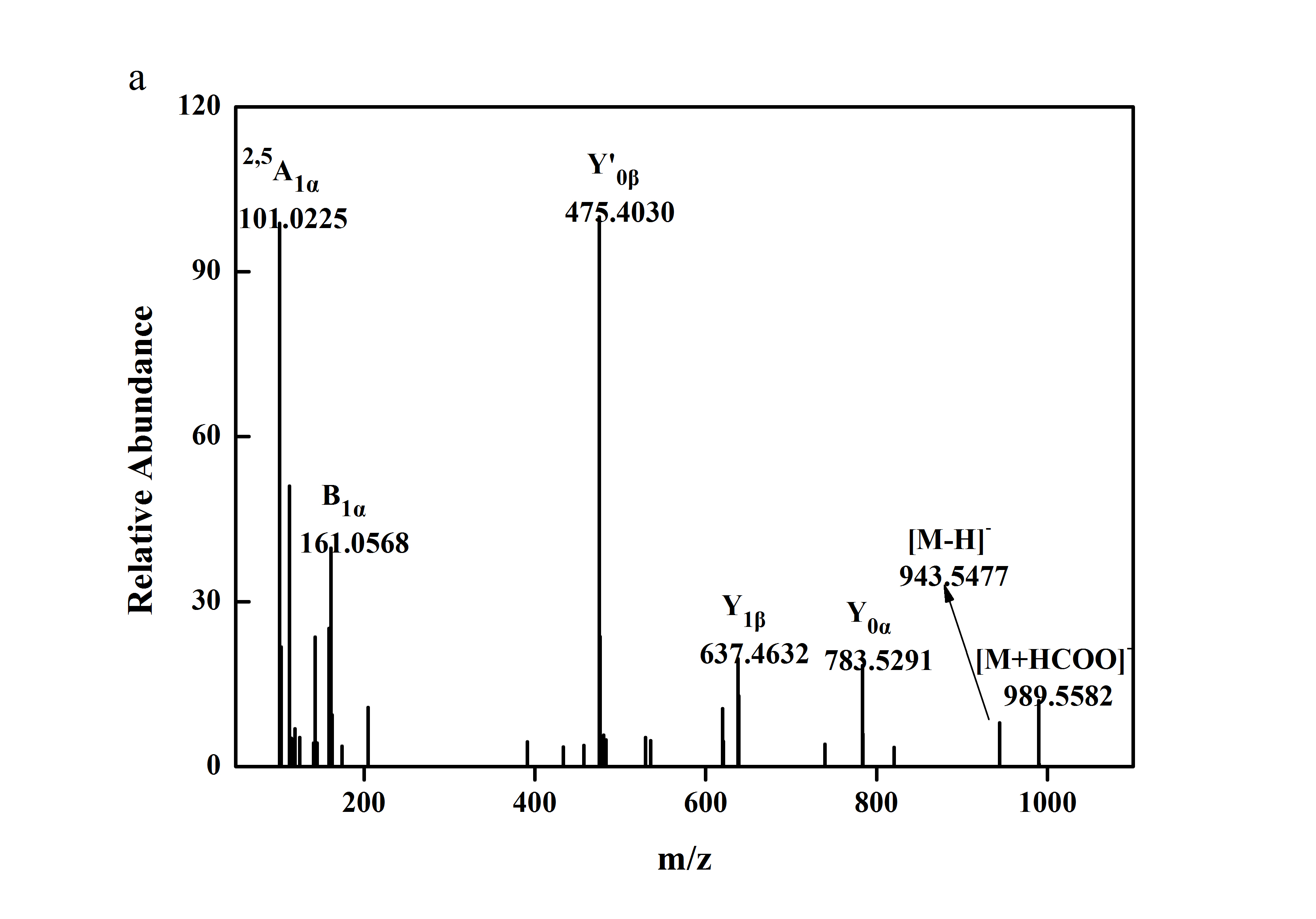


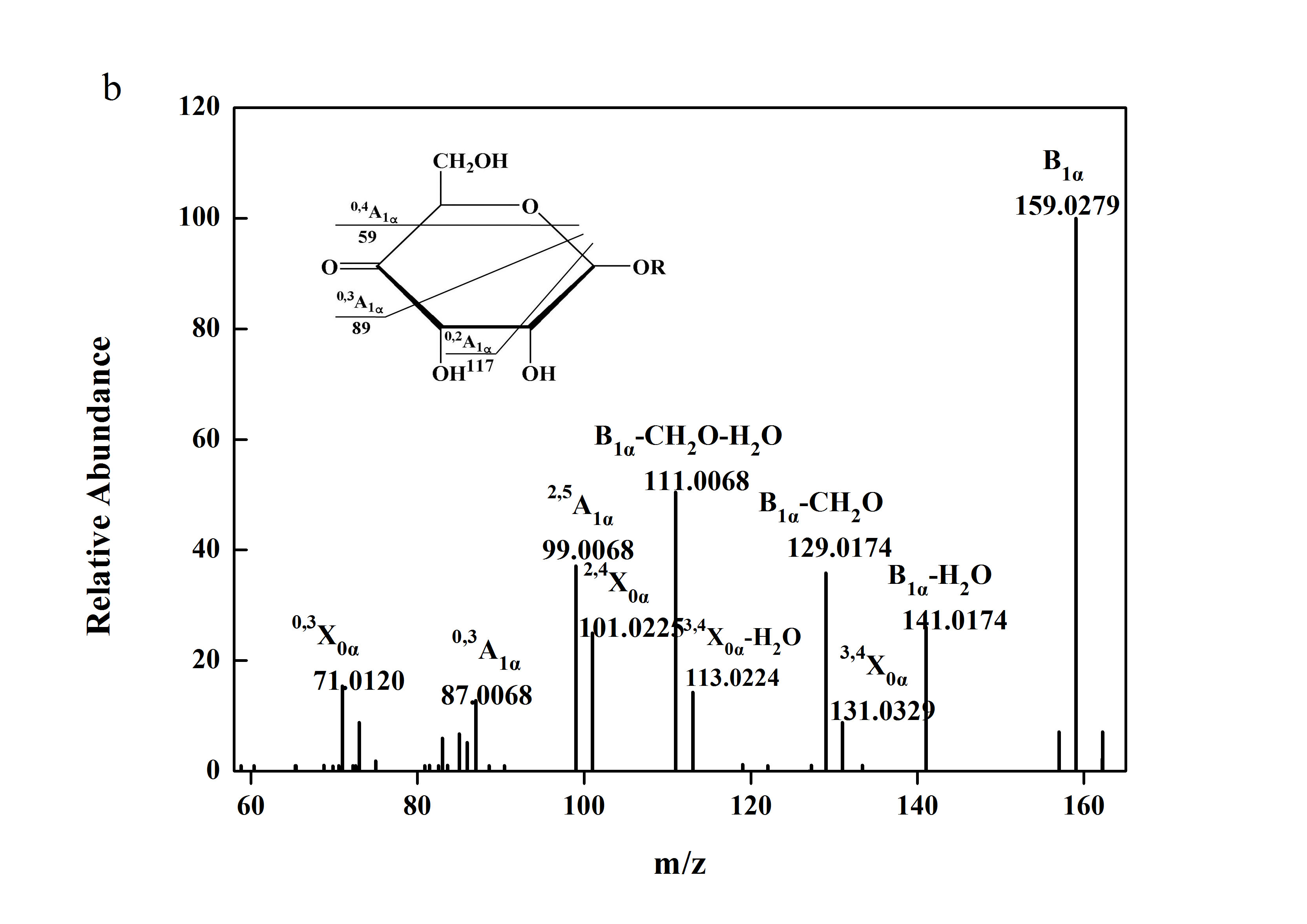
**Figure S4**. (a) MS/MS spectrum in negative ion mode of metabolite 3 using RRLC-Q-TOF MS. (b) MS/MS spectrum in negative ion mode of metabolite 3 using UHPLC-Q-Exactive Orbitrap HRMS.


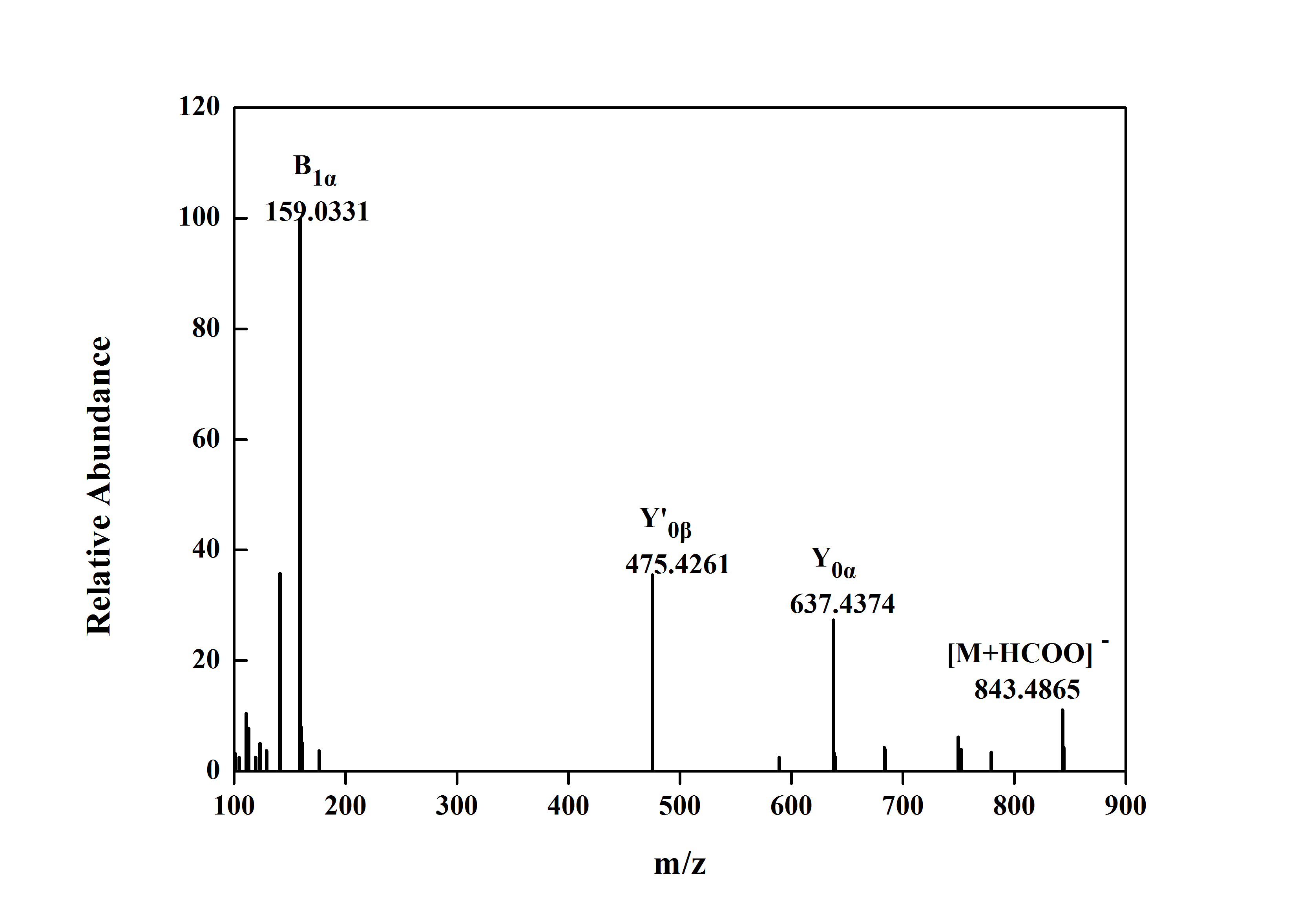


**Figure S5.** MS/MS spectrum in negative ion mode ofmetabolite 5 using RRLC-Q-TOF.


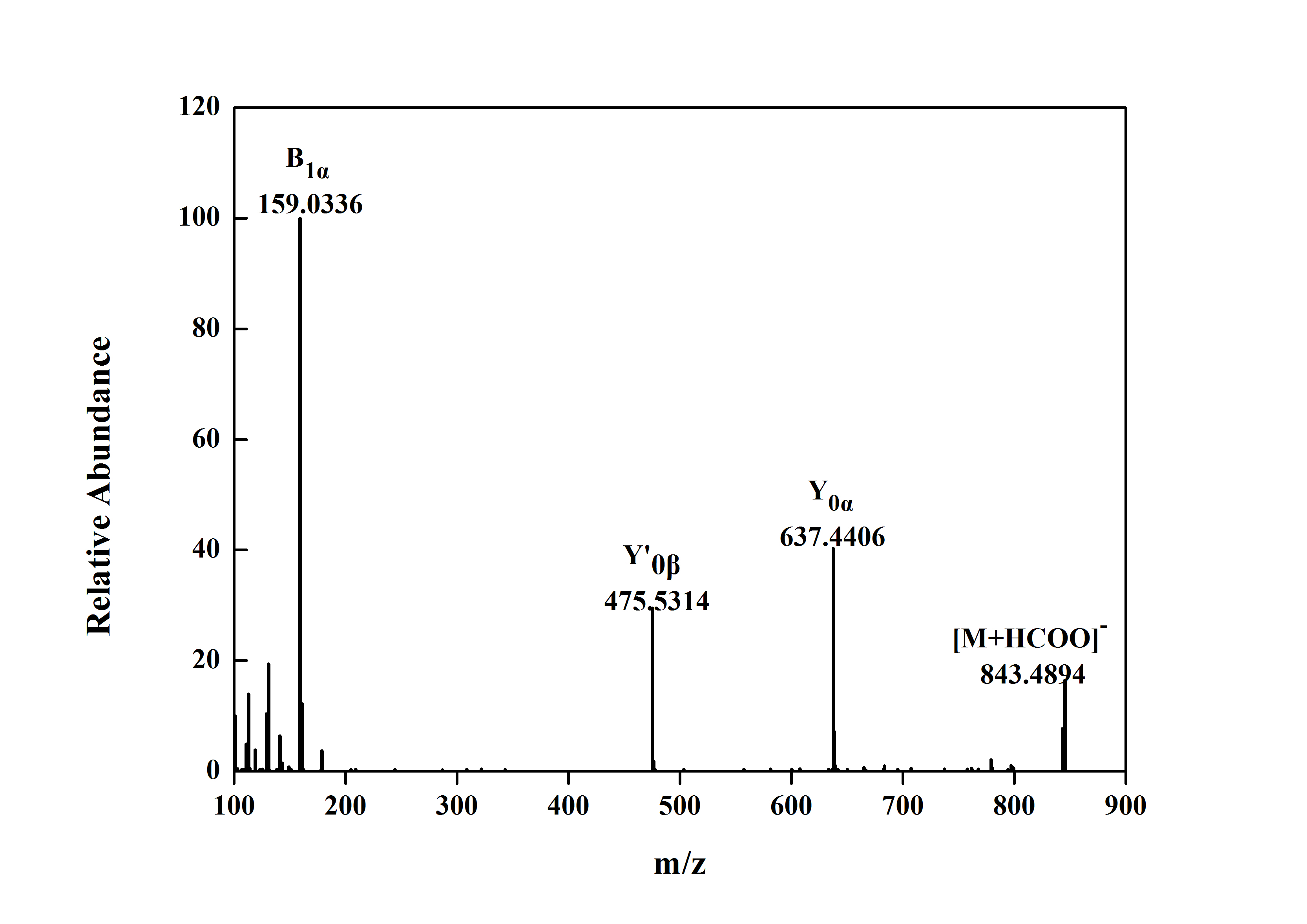


**Figure S6.** MS/MS spectrum in negative ion mode ofmetabolite 6using RRLC-Q-TOF.


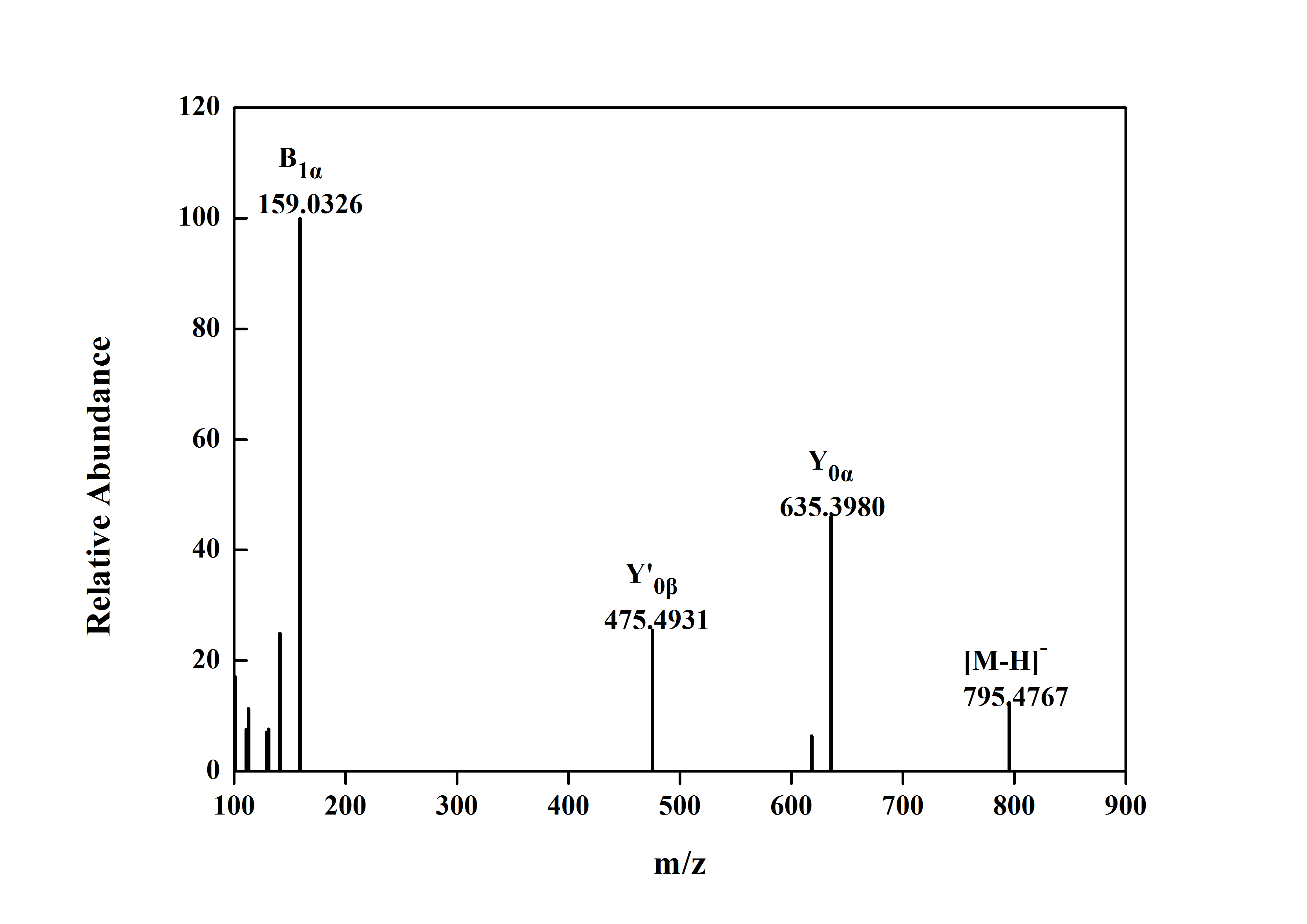


**Figure S7**. MS/MS spectrum in negative ion mode ofmetabolite 7 using RRLC-Q-TOF.


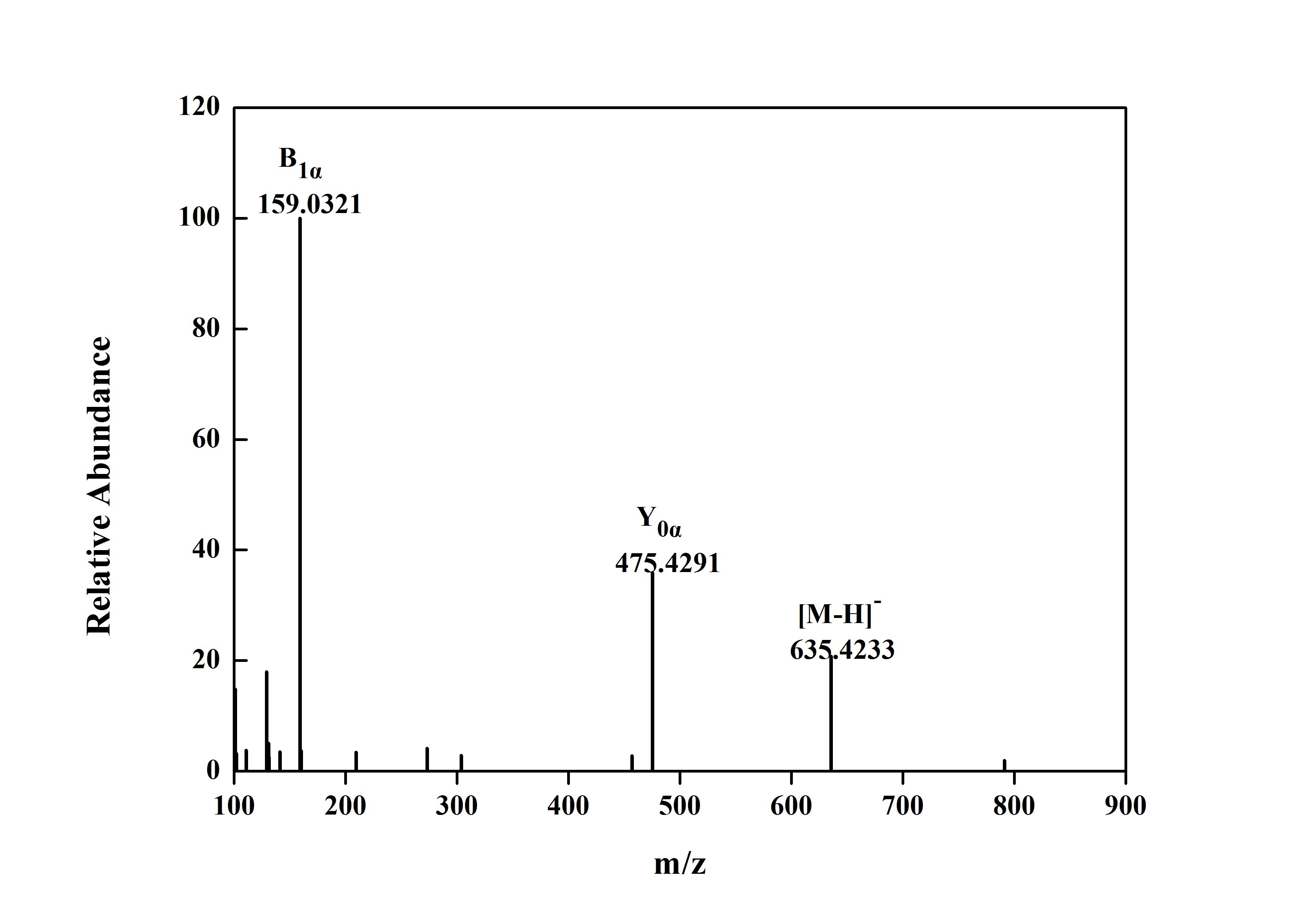


**Figure S8.** MS/MS spectrum in negative ion mode ofmetabolite 10 using RRLC-Q-TOF.


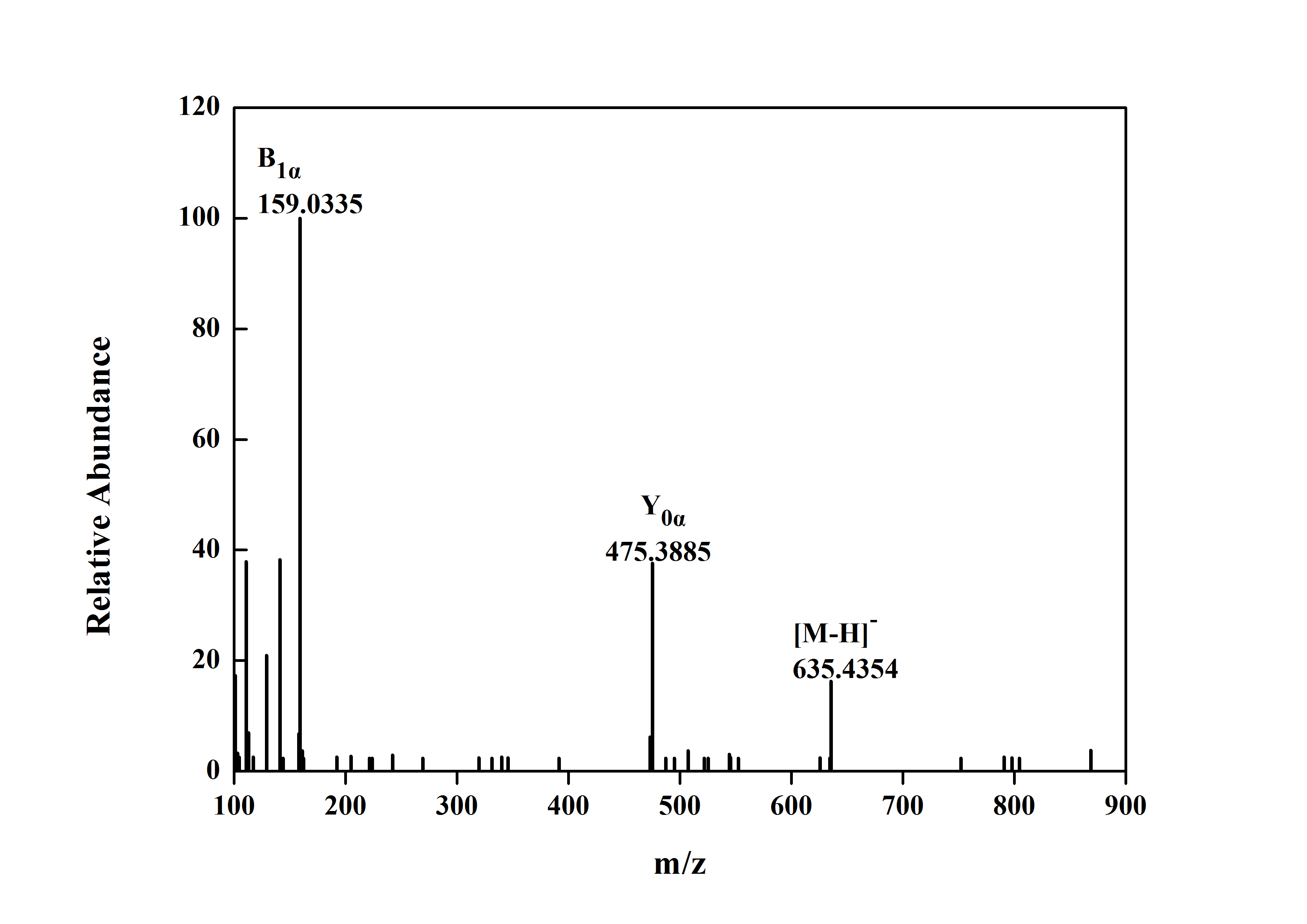


**Figure S9.** MS/MS spectrum in negative ion mode ofmetabolite 11 using RRLC-Q-TOF.


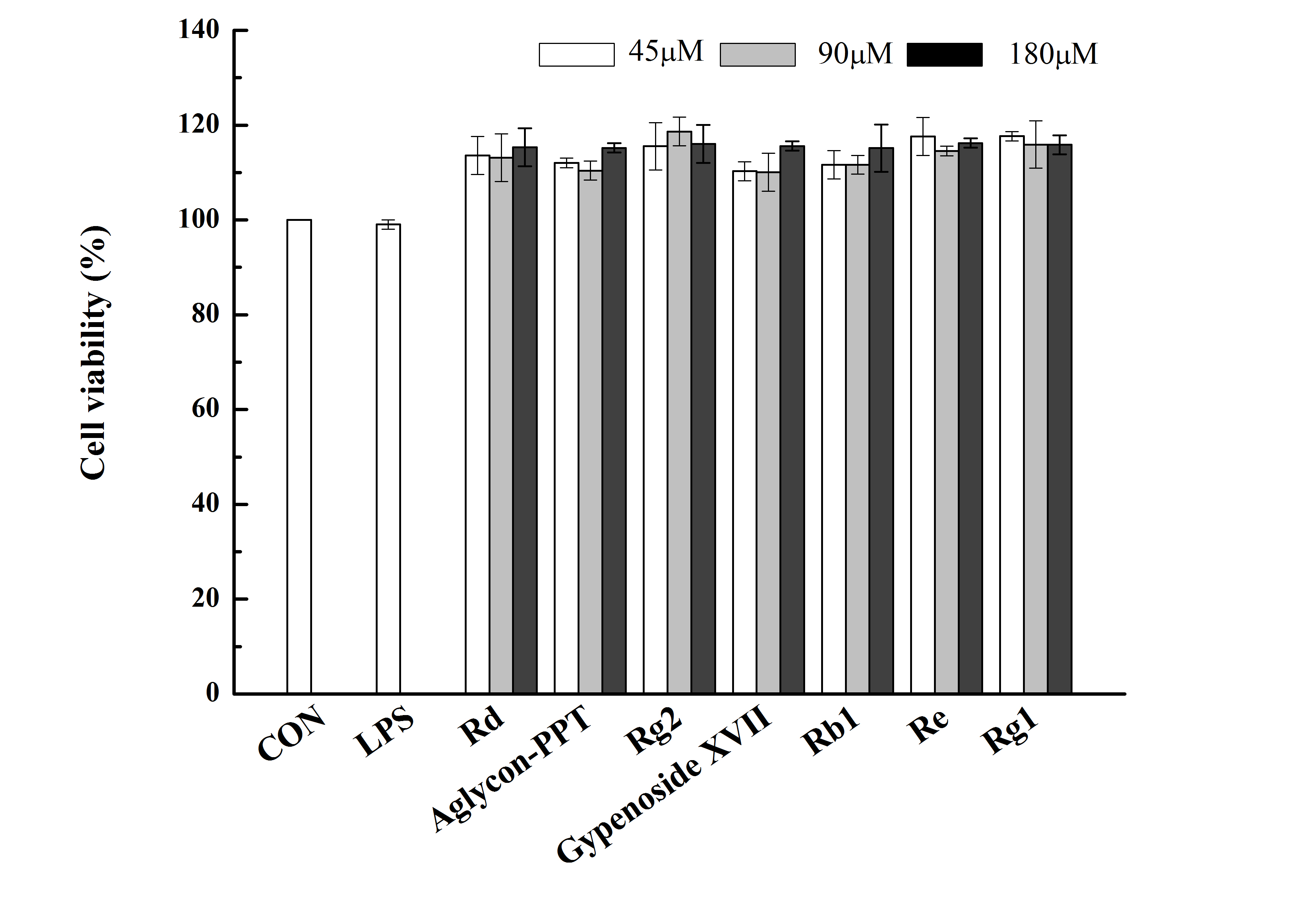


**Figure S10**. Effects of ginsenosides on cell viability of RAW 264.7 cells.RAW264.7 cells are incubated with ginsenosides at indicated concentrations for 24 h. Cell viability was measured by CCK-8 assay. Data are mean±SD (n=3). P>0.05 vs. the blank control.


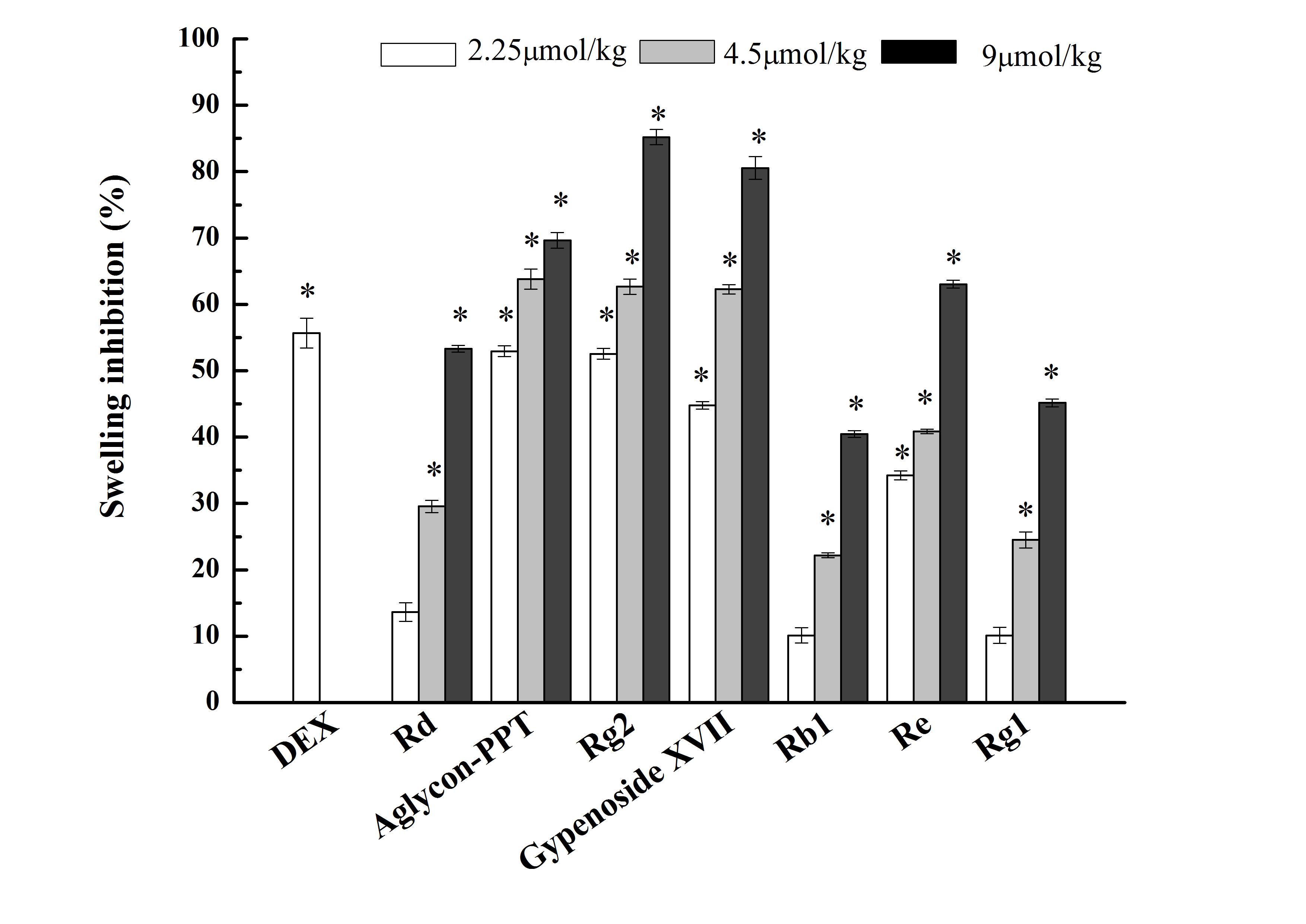


**Figure S11.** Effect of ginsenosides on mouse ear swelling response. The bar chart represented ear swelling inhibition rate of Dexamethasone group (1.5mg/kg) and ginsenosides groups (2.25, 4.5 and 9µmol/kg, respectively). *p<0.001 indicates significant difference from vehicle control.
